# Extract from a Memoir, Entitled "Enquiry into the Action of Some Vegetables upon the Spinal Marrow"

**Published:** 1809-08

**Authors:** M. Majendie


*Extract from a Memoir, entitled "Enquiry into the Action of some Vegetables upon the Spinal Marrow."*—Read before the French Institute 24th of April 1809; by M. MAJENDIE, M. D.

M. LECHENAUD, one of the naturalists who accompanied the expedition under Captain Baudin, having brought from Java and Borneo a great quantity of an extract tract from a vegetable called by the natives of Java, *Upas tienté,* with which they poison their arrows, Messrs. Majendie and Delille conceived the idea of determining by a series of experiments, the effects produced by this substance. M. Lechenaud himself is about to publish the description and critical analysis of this plant, which the learned author of the work upon the natural families of plants, has removed from the genus Strychnos and placed among the Apocynea.

With a view of imitating the process of the South Sea Islanders for poisoning their weapons, the authors of the memoir dipped in the extract of Upas some pieces of wood, of the size of a quill. When the poison was completely dry, they inserted the point of one of these pieces of wood into the thigh of a dog. For three minutes the animal appeared to suffer nothing except the pain of a common puncture; he was then seized with evident symptoms of general disease; almost all the muscles were convulsively contracted, the head was thrown backward, and the spine was rigidly bent in the same direction; the fore legs were lifted from the ground, from the curvature of the spine.

These convulsive motions lasted a moment only, and some seconds of tranquillity ensued, after which the convulsions recurred with double violence; the respiration became painful and quick; the convulsions were gradually stronger, and more frequent; and ended in a true tetanus. The dog fell upon his side, the legs became stiff, and stretched out; his breathing ceased, and the tongue and lips assumed a blue colour.

While the convulsions lasted, the state of circulation was ascertained by feeling the region of the heart; when a general tetanic stiffness was perceived for an instant only. The same effect was produced by touching the head, trunk, or limbs, and even the tail. About five minutes from the appearance of the first symptoms of poison, the animal was seized with a final tetanic convulsion, which was increased at intervals with violent shocks, and in two minutes it died. The brain seemed to have retained its functions during the strongest convulsions, and these functions only seemed weakened when the tetanus had almost entirely prevented respiration.

The same experiments, when repeated on a horse, six dogs, and three rabits, presented in all the same results, with this exception, that if the animal was vigorous and full grown, the convulsive fits were more frequent, more violent, and of longer duration; they recurred even

15 or 20 times before death. If the animal was young and feeble, it generally died in the third or fourth paroxysm.

On opening the bodies of animals killed by this poison, the muscular flesh with which it had come in contact, was of a yellowish brown colour, and the arterial and venous systems were filled with blood of a deep black colour. In order more precisely to ascertain the manner in which the Upas acts, Messrs. Majendie and Delille made several other experiments.

A small quantity having been dissolved in water, was injected into the cavity of the peritonæum of a dog. In about 20 seconds, the symptoms above described came on, and he died during the third convulsive attack.

Forty drops of Upas (dissolved as above), injected within the pleura of a horse, instantly produced tetanus and asphixia. He died during the second convulsion.

When injected into the cavities coated by mucous membranes, or mixed with food, the Upas was also absorbed, but much more slowly. It was half an hour before the symptoms appeared.

When applied to the serous and mucous membranes, no trace of local irritation was observed.

It was important to determine, whether this poison acted upon the nervous system through the medium of the circulation. In order to ascertain this, Messrs. Majendie and Delille injected 30 drops into the jugular vein of a strong and healthy horse. It was almost immediately seized with tetanus, and died in three minutes. Death always comes on more slowly if the poison has to take a long circuit before it reaches the spinal marrow.

If the poison be injected into one of the carotid arteries, the animal is in the first place violently agitated, and rolls about the moment the poison reaches the brain. These first symptoms go off in a short time, and are succeeded by those which seem to result from the action of the poison upon the spinal marrow.

If the spine is cut below the occipital bone after the inoculation or injection of the Upas into any part of the body, neither the convulsions nor tetanus are stopped by the operation, and they continue while the circulation also goes on. In this last experiment, weak animals die much sooner than such as are vigorous.

If, instead of cutting the marrow below the occiput, we disorganize it completely by inserting a piece of whalebone or metal into the vertebral canal, convulsions do not take place, or if they have already commenced, the instant the marrow is wounded, they cease in succession sion from the fore to the hind legs, in proportion as the instrument penetrates more deeply into the spinal marrow.

When the spine is cut across, and one of its extremities placed in contact with a small quantity of Upas, those parts only become convulsed which receive nerves from the portion of the marrow, upon which the poisonous substance acts.

The above are the chief phenomena observed by Messrs. Majendie and Delille in their numerous experiments. They evidently prove,

1st. That the extract of Upas acts in a particular manner upon the spinal marrow, in whatever way it is introduced into the system.

2d. That animals poisoned by this substance die of asphixia, which comes on when tetanus affects the muscles of the chest.

A great number of experiments also demonstrated to the authors of the memoir, that the Upas when inoculated or mixed with food in small doses, does not produce death, but merely the effects resulting from any other strong excitement of the spinal marrow.

May not this substance be usefully employed in some diseases which seem to arise from a weakness or defect of action in this part of the system? Such is the question with which Messrs. Majendie and Delille conclude their memoir.

